# Anthropogenic depletion of Iran’s aquifers

**DOI:** 10.1073/pnas.2024221118

**Published:** 2021-06-14

**Authors:** Roohollah Noori, Mohsen Maghrebi, Ali Mirchi, Qiuhong Tang, Rabin Bhattarai, Mojtaba Sadegh, Mojtaba Noury, Ali Torabi Haghighi, Bjørn Kløve, Kaveh Madani

**Affiliations:** ^a^Water, Energy and Environmental Engineering Research Unit, Faculty of Technology, University of Oulu, 90014 Oulu, Finland;; ^b^School of Environment, College of Engineering, University of Tehran, 1417853111 Tehran, Iran;; ^c^Department of Biosystems and Agricultural Engineering, Oklahoma State University, Stillwater, OK 74078;; ^d^Key Laboratory of Water Cycle and Related Land Surface Processes, Institute of Geographic Sciences and Natural Resources Research, Chinese Academy of Sciences, 100101 Beijing, China;; ^e^College of Resources and Environment, University of Chinese Academy of Sciences, 100049 Beijing, China;; ^f^Department of Agricultural and Biological Engineering, University of Illinois at Urbana–Champaign, Urbana, IL 61801;; ^g^Department of Civil Engineering, Boise State University, Boise, ID;; ^h^Science and Research Branch, Islamic Azad University, 1477893855 Tehran, Iran;; ^i^The Whitney and Betty MacMillan Center for International and Area Studies, Yale University, New Haven, CT 06511;; ^j^Department of Physical Geography, Stockholm University, SE-106 91 Stockholm, Sweden

**Keywords:** groundwater depletion, salinity, water resources management, water quality

## Abstract

Global groundwater assessments rank Iran among countries with the highest groundwater depletion rate using coarse spatial scales that hinder detection of regional imbalances between renewable groundwater supply and human withdrawals. Herein, we use in situ data from 12,230 piezometers, 14,856 observation wells, and groundwater extraction points to provide ground-based evidence about Iran’s widespread groundwater depletion and salinity problems. While the number of groundwater extraction points increased by 84.9% from 546,000 in 2002 to over a million in 2015, the annual groundwater withdrawal decreased by 18% (from 74.6 to 61.3 km^3^/y) primarily due to physical limits to fresh groundwater resources (i.e., depletion and/or salinization). On average, withdrawing 5.4 km^3^/y of nonrenewable water caused groundwater tables to decline 10 to 100 cm/y in different regions, averaging 49 cm/y across the country. This caused elevated annual average electrical conductivity (EC) of groundwater in vast arid/semiarid areas of central and eastern Iran (16 out of 30 subbasins), indicating “very high salinity hazard” for irrigation water. The annual average EC values were generally lower in the wetter northern and western regions, where groundwater EC improvements were detected in rare cases. Our results based on high-resolution groundwater measurements reveal alarming water security threats associated with declining fresh groundwater quantity and quality due to many years of unsustainable use. Our analysis offers insights into the environmental implications and limitations of water-intensive development plans that other water-scarce countries might adopt.

## Significance

Iran is facing a state of water bankruptcy that threatens its socioeconomic development and natural environments. Using an exceptionally rich measured groundwater dataset, we illustrate the extent and severity of Iran’s groundwater depletion and salinization problems during the 2002 to 2015 period, when the number of groundwater extraction points nearly doubled. Iran’s nonrenewable groundwater withdrawal was about 66 million m^3^ in 1965, which cumulatively grew to approximately 133 × 10^3^ million m^3^ in 2019. This increase is about 3.4 times the capacity of the famous Three Gorges Dam in China. Groundwater decline due to extensive overexploitation of nonrenewable groundwater and rising salinity levels are documented in almost all subbasins, pointing to dire, worsening water security risks across the country.

Groundwater is the backbone of water and food security in arid/semiarid areas, including Iran, with spatial and temporal changes due to natural surface water variability and scarcity. Groundwater provides about 60% of the total water supply in Iran (1), where agriculture is responsible for more than 90% of water withdrawal (2). Systematic groundwater extraction in Iran dates back at least two and a half millennia, when underground aqueducts known as “qanats” were excavated to transfer groundwater to the surface under the force of gravity (3). The Persian qanats that had facilitated development and agricultural production in Iran for thousands of years mostly dried up with technological advances and modernization of agriculture in the 20th century (4). Deep well drilling made groundwater overexploitation possible, while increased surface water damming and diversion reduced groundwater recharge, together drawing down groundwater tables (*SI Appendix*, Fig. S1) and making groundwater harvesting through historical qanats less feasible. Aggressive water resources development (1, 2, 5) to support the livelihood of over 80 million people and irrigate about 5.9 million ha of agricultural land heightened the pressure on groundwater. Iran’s water scarcity in the 21st century has been exacerbated by frequent droughts and climatic changes (2, 6). On average, more than half of the design capacity of Iran’s reservoirs was empty from 2003 to 2017 (7), increasing the reliance on groundwater. Consequently, Iran was ranked among the countries with the highest groundwater depletion rate in the 21st century, along with India, the United States, Saudi Arabia, and China (8).

Iran is grappling with acute water management problems and tensions (9, 10). Groundwater overdraft has contributed to a host of contemporary socioecological problems, including the drying up of wetlands, desertification, sand and dust storms, deteriorating water quality, and population displacement (10, 11). Land subsidence due to groundwater depletion is now a manmade hazard to vital infrastructure and residents in vulnerable plains (12). Further, declining groundwater tables have degraded groundwater quality due to natural processes such as saltwater intrusion (13–15). The increasing strain on rural livelihoods and mounting tensions among groundwater users exacerbate food and water security risks (16), and create sociopolitical issues related to the migration of rural populations to urban areas (17).

Groundwater assessments based on global models and remote sensing approaches have offered high-level characterizations of Iran’s groundwater resources (8, 18, 19). However, these investigations are limited by coarse spatial scales and large uncertainties due to lack of ground-truth data, hindering the detection of regional imbalances between renewable groundwater supply and human withdrawals. This study provides a statistical analysis of the major groundwater characteristics using a rich ground-based dataset (2002 to 2015) to determine the groundwater depletion and salinization in all 30 subbasins of Iran (*SI Appendix*, Fig. S2). The investigation of the temporal trend and spatial distribution of groundwater depletion and salinity provides valuable information for effective management of aquifers across Iran, and offers insights to other countries facing similar water security issues.

## Results and Discussion

**Fig. 1.**
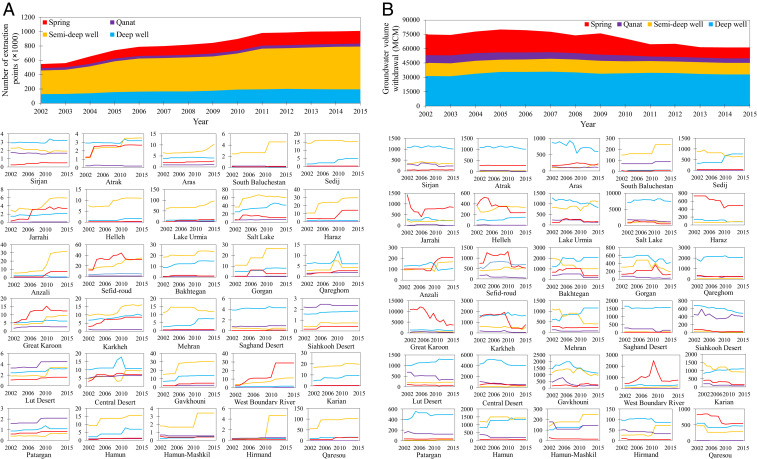
(*A*) Number of groundwater extraction points including deep wells (blue), semideep wells (yellow), qanats (purple), and springs (red). (*B*) Annual groundwater withdrawal from extraction points in Iran and in each of the 30 subbasins during 2002 to 2015. Total annual extracted groundwater volume decreased about 18% across Iran, mainly due to limited groundwater availability. Maximum groundwater withdrawal occurred during the 2005-to-2008 drought.

The number of groundwater extraction points has increased rapidly across Iran (Fig. 1*A*), disrupting the natural groundwater balance in many aquifers. The number of deep wells, semideep wells, qanats, and springs used to meet the increasing water demand rose by 52.4, 81.4, 22.2, and 221.7%, respectively, increasing the total number of withdrawal points by 84.9% from 2002 to 2015 (Fig. 1*A*). Semideep wells generally outnumber deep wells in most subbasins except those located in the Central Plateau and Qareghom major basins, where groundwater tables are very deep (up to 250 m). This situation may accelerate saltwater intrusion in the vicinity of inland saline lakes (e.g., Salt Lake, Jazmorian Wetland, and Bakhtegan Lake). Springs are dominant in the north and west (e.g., Sefid-roud, West Boundary River, and Great Karoon subbasins), where most perennial rivers flow. Also, there are a large number of qanats in the arid subbasins, including Siahkooh Desert, Central Desert, Lut Desert, and Patargan (Fig. 1*A*).

The total amount of groundwater withdrawal decreased from 74.6 billion cubic meters (km^3^) in 2002 to 61.3 km^3^ in 2015 (Fig. 1*B*) despite the increase in the number of extraction points (Fig. 1*A*). Two possible reasons exist: 1) growing strain on shallow aquifers causing groundwater depletion and reduced shallow groundwater yield and quality; and 2) potential effectiveness of groundwater monitoring, management, and conservation policies. Although in actuality the trend is affected by a combination of both these reasons, ground-based data indicate that many areas have reached the physical limits of renewable groundwater, suggesting that groundwater management programs have largely failed. Further, the operation times of deep and semideep wells increased by 17.2% (*SI Appendix*, Fig. S3), indicating an intentional effort to increase groundwater withdrawal. However, the amount of groundwater withdrawal from all sources declined (semideep wells: −12.3%; springs: −47.1%; qanats: −42.1%), except deep wells, which showed an increase in withdrawal (+5.4%) (Fig. 1*B*). This reflects that aggressive groundwater abstraction has persisted despite the government’s claimed groundwater regulation efforts. Over the same period, the total discharge from the groundwater nodes fell by about 51.0% (*SI Appendix*, Fig. S4). The share of deep and semideep wells in Iran’s total groundwater supply increased from 42.1 to 54.1% and from 18.7 to 20.0%, respectively. These increases were concurrent with a drop in the share of springs (28.4 to 18.3%) and qanats (10.8 to 7.6%) during 2002 to 2015 (Fig. 1*B*). The increased number of wells extracting water from deeper depths reduces the productivity of springs and qanats by drawing down the groundwater tables. This empirical evidence agrees with other groundwater studies carried out in different parts of Iran (e.g., refs. 1, 20, and 21).

**Fig. 2.**
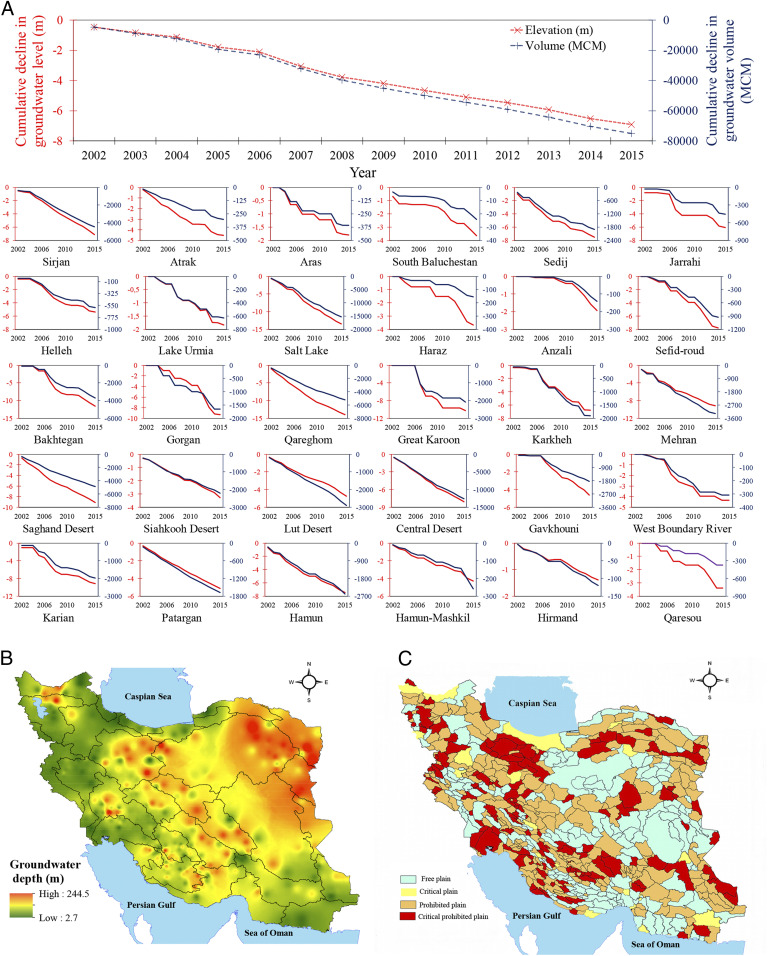
(*A*) Temporal trend of cumulative decline in groundwater resource volume and groundwater level. Both temporal trends show a continuous decline at national and subbasin scales. Salt Lake (Daryacheh Namak) and South Baluchestan subbasins experienced the largest and smallest annual average DGRV, respectively. Hirmand had the smallest annual average DGRL and Qareghom had the largest annual average DGRL. (*B*) Spatial distribution of average groundwater level based on the inverse distance weighting method using groundwater level data from 12,230 piezometers measured during the study period. The average groundwater level varied between 2.7 and 244.5 m. (*C*) Spatial distribution of “free plains” (215 plains), “critical plains” (31 plains), “prohibited plains” (236 plains), and “critical prohibited plains” (127 plains) across Iran as of 2018 (2). Free plains are areas where permits are issued for drilling new wells. Critical prohibited plains and prohibited plains refer to places where drilling new wells is banned except for potable water. Critical plains are projected to reach prohibited-plain status in the future.

The extensive groundwater withdrawal caused an average annual net depletion of about 5.4 km^3^ of nonrenewable groundwater (i.e., negative balance in aquifers) across Iran (Fig. 2*A* and *SI Appendix*, Table S1). Total nonrenewable groundwater withdrawal during the 14-y period of this study was about 75 km^3^. To understand the significance of this number, one must note Iran’s average total annual renewable water (both surface water and groundwater), which official water authorities claim to have declined from 130 km^3^ to less than 100 km^3^ due to anthropogenic and climatic changes (10). According to available historical data from the Iran Water Resources Management Company (IWRMC), the country’s nonrenewable groundwater withdrawal (about 66 million m^3^; MCM) was first reported in 1965. The cumulative depletion of Iran’s fossil groundwater storage is estimated to have grown to ∼133 km^3^ by 2019 (22). This amount of depletion is about 3.4 times the storage volume of the reservoir formed by the famous Three Gorges Dam in China, putting in perspective the magnitude of nonrenewable groundwater depletion in Iran. All the subbasins experienced a net decline in groundwater resource volume (DGRV) as cumulatively shown in Fig. 2*A*. Highly populated Salt Lake and less populated South Baluchestan subbasins experienced the largest (>−1,089 MCM/y) and smallest (up to −1.7 MCM/y) annual average DGRV, respectively (Fig. 2*A* and *SI Appendix*, Table S1). In a larger spatial unit, Qareghom and Lake Urmia major basins showed the largest and smallest annual average DGRV normalized by basin area during the study period, respectively (*SI Appendix*, Fig. S5).

Nonrenewable groundwater extraction caused a cumulative decline in groundwater resource level (DGRL), averaging about 49 cm/y across the country (Fig. 2*A* and *SI Appendix*, Table S1). Declines in groundwater level for individual subbasins illustrate the range of DGRL between about 10 cm/y in Hirmand and 100 cm/y in Qareghom. Also, Qareghom and Lake Urmia major basins experienced the largest and smallest annual average DGRL during the study period, respectively (*SI Appendix*, Fig. S5). At a finer spatial resolution, the average groundwater level recorded at 12,230 piezometers scattered across the country (*SI Appendix*, Fig. S6) from 2002 to 2015 varied between 2.7 and 244.5 m (Fig. 2*B*), indicating the deepest groundwater tables in the Qareghom major water basin. While no explicit data were available about the groundwater recharge at country scale, the results presented for annual net decline in groundwater volume and level, namely DGRV and DGRL (Fig. 2*A* and *SI Appendix*, Fig. S5 and Table S1), demonstrate a negative balance between groundwater discharge and recharge in all subbasins and at country level. The extensive groundwater table decline during the last five decades has increased the number of “prohibited plains,” where drilling of new wells is banned (except for potable water) (*SI Appendix*, Fig. S7). Only 215 (35%) of Iran’s 609 plains are currently classified as “free plains,” where the government issues permits for drilling new wells (*SI Appendix*, Fig. S2*C*). The growing number of prohibited plains is a compelling sign that regulatory policies for managing the strained groundwater resources are inadequate.

**Fig. 3.**
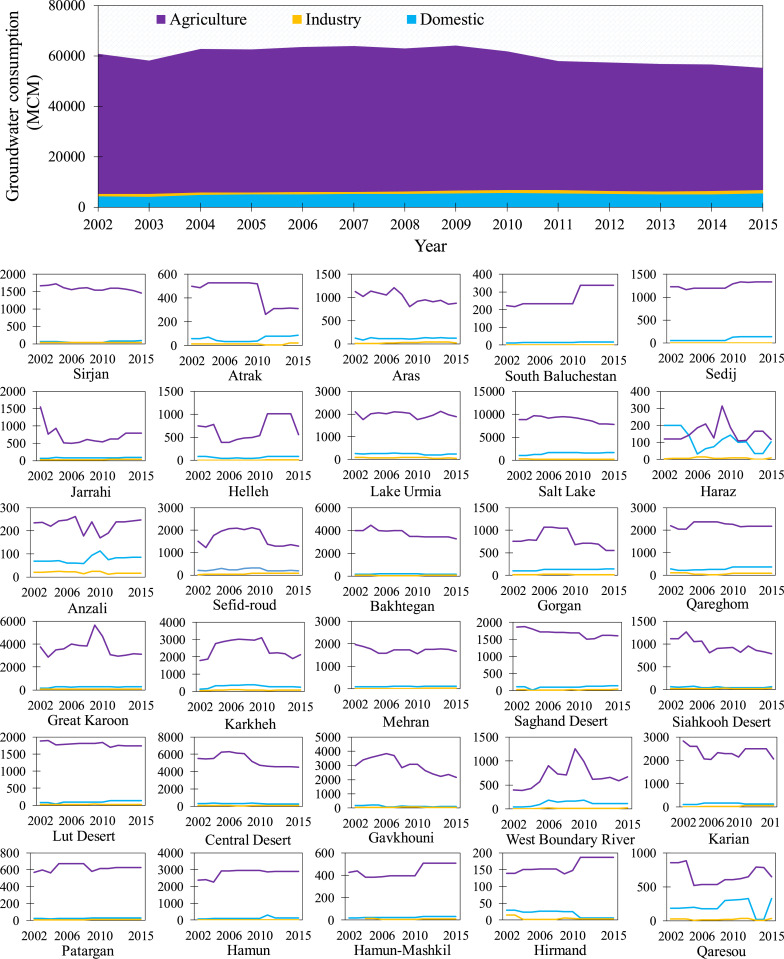
Annual average groundwater consumption in Iran’s agricultural (purple), domestic (blue), and industrial (yellow) sectors across the country and in each subbasin during 2002 to 2015.

Groundwater consumption decreased from 60.7 km^3^ in 2002 to 55.2 km^3^ in 2015 (Fig. 3). Because of heavily subsidized energy and water, agricultural water consumption is effectively only curtailed due to surface water and fresh groundwater shortages (1), leading to a widespread overshoot of the renewable water supply capacity, namely “water bankruptcy” (10), with some environmental damages (e.g., dried wetlands, soil erosion, and desertification) that are irreversible in a short time frame. The share of the agricultural sector in groundwater consumption decreased from 91.0% in 2002 (55.3 km^3^) to 87.6% in 2015 (48.3 km^3^), whereas domestic and industrial groundwater consumption increased from 7.2 (4.36 km^3^) to 10.2% (5.60 km^3^) and 1.8 (1.10 km^3^) to 2.3% (1.26 km^3^), respectively. During this period, the annual change in agricultural groundwater consumption was negative in most subbasins (Fig. 3), mainly due to reduced good-quality groundwater yield. The Salt Lake subbasin had the maximum decrease in groundwater consumption for both agricultural (∼−72.9 MCM/y) and industrial (about −8.0 MCM/y) sectors and the maximum increase in domestic groundwater consumption (∼41.6 MCM/y). According to Iran’s latest population census in 2016, about 26% of the country’s population resided in the Salt Lake subbasin. Rapid population growth coupled with surface water shortages due to severe and prolonged droughts in the Salt Lake subbasin prompted a gradual replacement of surface water with groundwater to provide secure domestic water. Our findings reveal an increasing ratio of groundwater consumption to groundwater withdrawal during the study period (*SI Appendix*, Fig. S8). Increase in the consumptive use of groundwater, which reduces return flows and groundwater recharge, has major environmental and water management implications. This trend can be mainly attributed to the transfer of historical agricultural groundwater share to the domestic and industrial sectors as well as the technological advancement of farming practices and increased agricultural water-use efficiency (e.g., irrigation efficiency improvements). Reduced recharge from human uses combined with reduced natural recharge during the dry years in the study period (2) also explains why shallow groundwater consumption has declined during the study period.

**Fig. 4.**
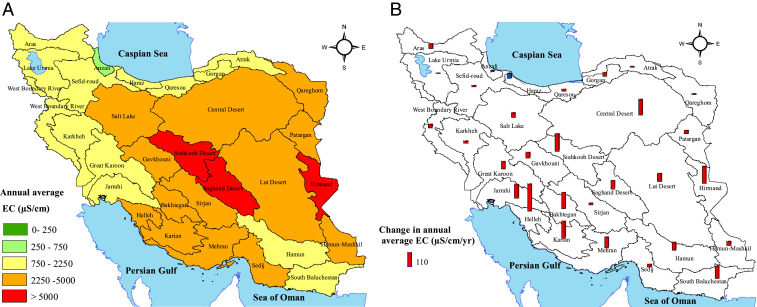
Spatial distribution of annual average EC (*A*) and changes in annual average EC from 2002 to 2015 (*B*). Measured annual average EC indicates very high salinity hazard (EC more than 2,250 µS/cm) in 16 out of 30 subbasins. Also, changes in annual average EC were positive (i.e., deteriorating; red bars) in almost all subbasins (except for the water-rich Anzali and Haraz subbasins; blue bars).

The average electrical conductivity (EC) recorded at the 14,850 observation wells distributed across the country (*SI Appendix*, Fig. S9) fluctuated between 34.6 and 36,307.8 µS/cm during 2002 to 2015 (*SI Appendix*, Fig. S10). The annual average EC in central and eastern Iran was higher than in other parts (Fig. 4*A*). More than half of the subbasins (16 out of 30) indicated a “very high salinity hazard,” that is, 2,250 ≤ EC < 5,000 µS/cm (Fig. 4*A*), according to US Salinity Laboratory (USSL) guidelines for the classification of irrigation water (23). These subbasins have effectively reached marginal to low-quality groundwater, which is only suitable for irrigating salt-tolerant plants. Only the Anzali subbasin in northern Iran had low to medium salinity based on annual average EC (Fig. 4*A*). Further, the annual maximum EC in 20 out of 30 subbasins exceeded 5,000 μS/cm, the upper threshold in the USSL classification for irrigation of salt-tolerant crops in light soils (*SI Appendix*, Fig. S11). The highest EC level was recorded in the Mehran and Helleh subbasins (> 32,000 µS/cm) adjacent to the Persian Gulf, implying saltwater intrusion into the coastal aquifers. In three subbasins (Hirmand, Saghand Desert, and Siahkooh Desert), annual average EC exceeded the 5,000 μS/cm threshold, meaning that groundwater is not generally suitable for irrigation. Annual average EC increased in most parts of Iran (i.e., 28 out of 30 subbasins), especially in the central, eastern, and southwestern regions (Fig. 4*B*). Based on the change of annual average EC, the Siahkooh Desert (135.5 µS⋅cm^−1^⋅y^−1^), Karian (133.3 µS⋅cm^−1^⋅y^−1^), and Hirmand (132.1 µS⋅cm^−1^⋅y^−1^) subbasins showed the highest rates of salinity-related groundwater quality deterioration (Fig. 4*B*). Increasing EC in irrigation water can reduce soil quality and agricultural production (24) and accelerate desertification (25). Meanwhile, groundwater quality slightly improved in terms of the rate of annual average EC in a few subbasins located in the northern parts of Iran (e.g., the water-rich Haraz and Anzali, where the rate of annual average EC decreased by 40.6 and 9.2 µS⋅cm^−1^⋅y^−1^, respectively) (Fig. 4*B*).

The in situ groundwater quantity and quality data allowed a robust assessment of the fresh groundwater decline in vast areas of Iran. The combination of fresh groundwater depletion and salinization indicates Iran’s alarming water and environmental security risks with critical implications for food security through jeopardizing salt-intolerant crops. Iran’s political economy relies on water and agriculture (2) and groundwater bankruptcy [the end state of a coupled human–nature process (26) in which the groundwater supply–use gap is huge and some of the resulting damages of groundwater depletion (e.g., land subsidence and sinkholes) are irreversible in the short run]. The water security threat can cause unemployment, migration, and other major socioeconomic problems. The country’s water-dependent development path (9) in light of the compelling signs of water bankruptcy is bound to increase competition and disputes over depleting groundwater resources to buffer surface water variability and shortage (10). The number of Iran’s prohibited and critically prohibited plains will continue to rise if the current trend of groundwater table decline persists throughout the 21st century under the status quo water management and dwindling renewable water.

## Materials and Methods

### Study Area and Data.

Iran’s territory has been divided into 6 major basins, 30 subbasins (*SI Appendix*, Fig. S2), and 609 plains. Iran’s aquifers consist of 1) karstic formations located in the west, which recharge the perennial rivers such as Dez, Karoon, and Karkheh; 2) alluvial deposits such as the Tehran-Karaj aquifer; and 3) coastal aquifers along the shorelines of the Persian Gulf, Sea of Oman, Caspian Sea, and inland saline lakes. The aquifers occupy about 260,000 km^2^ (i.e., 16%) of Iran’s territory (*SI Appendix*, Fig. S2).

Iran’s national groundwater-monitoring system was established in the 1970s (22), and it covered 647 of the 758 identified aquifers in 2016. The historical data (2002 to 2015) used in this study were obtained from the IWRMC, the highest-level government organization responsible for collecting and disseminating water-related data as a subsidiary of Iran’s Ministry of Energy. The 2002-to-2015 interval was chosen as the study period since the IWRMC does not provide data with a similar spatial and temporal resolution for later years. The data were analyzed for each subbasin and basin and the country (*SI Appendix*, Section S1). The available dataset provides annual aggregation of measured and estimated groundwater data from sampling points to represent conditions at subbasins, basins, and ultimately the country. Groundwater quantities (count of extraction points, groundwater discharge, withdrawal, and consumption) cover both permitted and unpermitted wells. Unpermitted wells play an important role in Iran’s groundwater since they account for about 330,000 out of some 800,000 wells that are now operational in the country (22). Specifically, the dataset includes:

1) Measured data: EC measurements in observation wells, inventory of extraction points (semideep and deep wells, qanats, and springs) and their discharge, withdrawal and consumption, and groundwater table measurements at piezometers. The extraction point data are fully measured based on field inventories and sampling campaigns on approximately a 5-y cycle (in both wet and dry periods). The same measurements as the 5-y field campaigns are conducted twice a year (during both wet and dry periods) at a small but representative set of selected extraction points in different plains (27). Piezometer data are recorded continuously on a monthly basis while observation wells are typically monitored seasonally, twice a year, or annually.
2) Annual estimates: estimated data for unmeasured extraction points based on measured data from designated extraction point intraannual monitoring; and estimates of DGRV and DGRL based on estimated aquifer characteristics (i.e., aquifer area and specific yield) and measured groundwater table in piezometers.

### Data Uncertainty.

The presented analyses and results rely heavily on the quality of observed groundwater data. Poor information on aquifer characteristics (e.g., aquifer type and specific yield) that require extensive and costly geophysical explorations diminishes the ability to fully represent groundwater systems. The IWRMC performs a six-step data quality assurance process to verify the accuracy of measured data through compliance with available national guidelines, cross-examination of field campaigns through repetition, and cross-validation of water balance at regional and national levels (*SI Appendix*, Sections S1 and S2). After completing field measurements, sampling is repeated for a fraction of extraction points, observation wells, and piezometers within each plain to detect possible discrepancies and prescribe additional monitoring campaigns, if necessary. Any detected errors are resolved at the local level through an additional repetition of measurements at a prespecified fraction of samples for further cross-examination of field data. Subsequently, the reported field data undergo cross-validation at the regional and national levels based on water balance analyses, considering historical trends in regional groundwater and surface water (*SI Appendix*, Sections S1 and S2). The combination of bottom-up and top-down data quality assurance procedures adopted by the IWRMC helps reduce data uncertainties. However, common limitations, simplifying assumptions, and uncertainties in groundwater quantity and quality assessments (28), especially in data-poor regions at relatively large scales such as Iran, introduce uncertainty into measured and estimated groundwater data. Thus, the results presented herein should be viewed in light of four major sources of uncertainty:

1) Potential systematic errors—by instruments and/or operators—in the measured data.
2) Inference uncertainties in annual estimates. Two general data categories affected by this source of uncertainty include 1) estimated data for unmeasured extraction points based on intraannual/annual measurements of designated sites; and 2) estimated DGRV and DGRL. In addition to uncertainties in aquifer characteristics, the DGRL and DGRV estimates are affected by siting of piezometers in the monitoring network. Piezometers are often sited based on general characteristics of the plain rather than individual aquifers, diminishing their ability to fully represent the groundwater system.
3) Uncertainty in aggregation of measured and estimated groundwater data. Measured and estimated groundwater data from extraction points (except for their count), observation wells, and piezometers are aggregated based on the Thiessen method for water quantity and quality parameters (see IWRMC guidelines in *SI Appendix*, Section S1) to represent conditions at larger spatial scales, namely subbasins, basins, and the country. Human errors and algorithmic deficiencies can impact such aggregation of data.
4) Partial coverage of the groundwater-monitoring network. The groundwater-monitoring network currently covers 85.5% of the aquifers. In the remaining 14.5% of the aquifers, exploitation wells with limited operation time are typically used for groundwater sampling. Given potential differences between groundwater measurements at piezometers/observation wells and exploitation wells, the data sampled in these aquifers are associated with some degree of uncertainty.

## Supplementary Material

Supplementary File

## Data Availability

All study data are included in the article and/or supporting information.
