# Supplementary material for: Anthropogenic depletion of Iran’s aquifers
Source: Proc Natl Acad Sci U S A. 2021 Jun 14;118(25):e2024221118. doi: 10.1073/pnas.2024221118 (PMC8237601; doi:10.1073/pnas.2024221118)
Supplement: Supplementary File [file pnas.2024221118.sapp.pdf]

PNAS
www.pnas.org

Supplementary Information for

Anthropogenic Depletion of Iran's Aquifers

Roohollah Noori[a,1], Mohsen Maghrebi[b], Ali Mirchi[c], Qiuhong Tang[d,e], Rabin Bhattarai[f], Mojtaba Sadegh[g], Mojtaba Noury[h], Ali Torabi Haghighi[a], Bjørn Kløve[a], Kaveh Madani[i,j].

[a]Water, Energy and Environmental Engineering Research Unit, Faculty of Technology, PO Box 4300, 90014 University of Oulu, Finland, [b]School of Environment, College of Engineering, University of Tehran, 1417853111 Tehran, Iran, [c]Department of Biosystems and Agricultural Engineering, Oklahoma State University, 111 Agricultural Hall, Stillwater, Oklahoma 74078, USA, [d]Key Laboratory of Water Cycle and Related Land Surface Processes, Institute of Geographic Sciences and Natural Resources Research, Chinese Academy of Sciences, 100101 Beijing, China, [e]College of Resources and Environment, University of Chinese Academy of Sciences, 100049 Beijing, China, [f]Department of Agricultural and Biological Engineering, University of Illinois at Urbana-Champaign, Urbana, IL 61801, USA, [g]Department of Civil Engineering, Boise State University, 1910 University Drive, Boise, USA, [h]Islamic Azad University, Science and Research Branch, 1477893855 Tehran, Iran, [i]The Whitney and Betty MacMillan Center for International and Area Studies, Yale University, CT 06511 New Haven, USA, [j]Department of Physical Geography, Stockholm University, SE-106 91 Stockholm, Sweden.

[1]To whom correspondence may be addressed. Roohollah Noori

Email: roohollah.noori@oulu.fi (roohollahnoori@gmail.com)

Digital Object Identifier (DOI): https://doi.org/10.1073/pnas.2024221118

This PDF file includes:

- **Sections**

  Section S1. Description of the Iran Water Resources Management Company's (IWRMC) Quality Assurance/Control (QA/QC) Approach.

  Section S2. Data Quality and Confidence in the Data.

- **Figures**

  Figure S1. Changes in average groundwater table (1965-2001) and extracted groundwater volume (1974 to 2001) in Iran.

  Figure S2. Major water basins, sub-basins, and aquifers across Iran. Major water basins are: (i) Central Plateau, (ii) Persian Gulf and Sea of Oman, (iii) Caspian Sea, (iv) Eastern Boundary, (v) Lake Urmia, and (vi) Qareghom. Sub-basins are: (i-1) Salt Lake (Daryacheh Namak), (i-2) Gavkhouni (Gavkhuni), (i-3) Bakhtegan, (i-4) Sirjan, (i-5)

Hamun, (i-6) Lut Desert, (i-7) Central Desert, (i-8) Siahkooh Deseart, (i-9) Saghand Desert, (ii-1) West Boundary River, (ii-2) Karkheh, (ii-3) Great Karoon (Karoun or Karun), (ii-4) Jarrahi (Jarahi), (ii-5) Helleh, (ii-6) Karian, (ii-7) Mehran, (ii-8) Sedij, (ii-9) South Baluchestan, (iii-1) Aras, (iii-2) Sefid-roud, (iii-3) Haraz, (iii-4) Qaresou, (iii-5) Gorgan, (iii-6) Atrak, (iv-1) Patargan, (iv-2) Hirmand, (iv-3) Hamun-Mashkil, (v-1) Lake Urmia, and (vi-1) Qareghom. Aquifers underly about 260,000 km2 (i.e., 16 %) of Iran's area.

Figure S3. Operation time (in hour) for semi-deep wells (yellow), deep wells (blue), and all wells (purple) in Iran and in each sub-basin during 2002-2015.

Figure S4. Groundwater discharge (m^3/s) from deep wells (blue), semi-deep wells (yellow), qanats (purple), and springs (red), in Iran and in each sub-basin during 2002-2015.

Figure S5. Annual cumulative decline in groundwater resource volume (DGRV) and level (DGRL) in six major water basins across Iran from 2002 to 2015.

Figure S6. Spatial distribution of 12,230 piezometers in Iran.

Figure S7. Increase in the number of "prohibited plains" in Iran (where drilling of new wells is banned except for potable water) due to extensive groundwater table decline during the last five decades.

Figure S8. Average ratio of groundwater consumption to groundwater withdrawal across Iran during 2002–2015.

Figure S9. Spatial distribution of 14,850 observation wells for electrical conductivity (EC) measurement in Iran.

Figure S10. Spatial distribution of average electrical conductivity (EC) in Iran based on inverse distance weighting method using groundwater EC data from 14,850 observation wells. The average groundwater EC varied between 34.6 µS/cm and 36,307.8 µS/cm.

Figure S11. Spatial distribution of annual maximum electrical conductivity (EC) in sub-basins during 2002–2015. The maximum EC in 20 sub-basins exceeded 5,000 µS/cm, which is the upper threshold in the USSL classification for irrigation of salt-tolerant crops in light soils. The highest EC level was recorded in Mehran and Helleh sub-basins (> 32,000 µS/cm) adjacent to the Persian Gulf, indicating likely saltwater intrusion into the coastal aquifers.

- **Table**

Table S1. Annual decline in groundwater resources volume (DGRV) and level (DGRL) in each sub-brain during 2002-2015.

- **References**

Section S1. Description of the IWRMC's Quality Assurance/Control (QA/QC) Approach

The Iran Water Resources Management Company (IWRMC) implements a six-step QA/QC process at three levels (i.e., local, regional, and national) prior to releasing the data. The first three steps ensure the accuracy of monitoring campaigns. The subsequent three steps aim at verifying data quality by applying water balance validations at regional and national offices as described below.

- Step 1- Groundwater Measurement and Analysis: Regional water authorities usually manage field measurement campaigns in each plain using external consulting services with required expertise and local knowledge of the study area. All instruments (mostly digital and some analog) are calibrated before each monitoring/sampling campaign to avoid systematic errors that introduce uncertainty in the measured data. Groundwater monitoring and data analyses are compliant with national guidelines (e.g., 1-9) that are aligned with international protocols, guidelines and suggestion to ensure data accuracy and reduce uncertainty in field tests.

- Step 2- Repeated Sampling: After completing field measurements at groundwater extraction points (deep and semi deep wells, springs, and qanats), observation wells, and piezometers within each plain, the consultant repeats sampling for a fraction of measurements at the request of the regional water authority or their representative (third-party consultant) that oversees the field campaign to double-check the sampling to detect possible discrepancies and prescribe additional field campaigns to resolve the discrepancies. Please see IWRMC (8) for more information about the criteria and procedures for selecting groundwater extraction points, observation wells, and piezometers for sampling repetition.

- Step 3- Cross-examination of Field Measurements: The regional water authority or their designated third-party consultant conducts cross-examination of the selected fraction of extraction points, observation wells, and piezometers. Any detected errors in the data are resolved at the local level at the completion of this step.

- Step 4- Regional Water Balance Validation by the Regional Synthesis Group: The reported field data are checked by a Synthesis Group at each regional water authority. The Synthesis Group performs a water balance validation using measured groundwater data along with available surface water data. The approved groundwater data are transferred to the national IWRMC office for further scrutiny with respect to the requirements of regional water balance validations performed at the country level.

- Step 5- Regional Water Balance Validation by IWRMC's Bureau of Groundwater Research: The regional water balance approach is also performed independently at the IWRMC's Bureau of Groundwater Research for cross-validation of the data reported by regional water authorities. Further, the data are cross-validated with respect to historical trends in regional groundwater and surface water data to flag any inconsistencies (e.g., sharp rises or declines) due to erroneous data.

- Step 6- Regional Water Balance Validation by IWRMC's Synthesis Studies: The last step of the QA/QC approach is an additional cross-validation of the regional water balance performed by the IWRMC's Synthesis Studies. The data are cleared for release at the end of this step.

Section S2. Data Quality and Confidence in the Data

All monitoring data used in this research were collected under direct supervision of the IWRMC. The IWRMC has embedded various checks and balances in its QA/QC approach to ensure the data can be trusted for official water resources planning and management activities. We did not implement independent QA/QC procedures except for the EC data. We used total dissolved solids (TDS) measured at nearly 15,000 observation wells that are exclusively monitored for water quality, to check the quality of measured EC data at the corresponding observation well. Our investigation showed that almost all the measured EC data matched the total dissolved solids with correlation coefficients varying between 0.59 and 0.98. Thus, the data used in this study are deemed reliable notwithstanding various limitations and uncertainties associated with groundwater quantity and quality monitoring efforts and simplifying assumptions for groundwater assessment at various spatial scales.

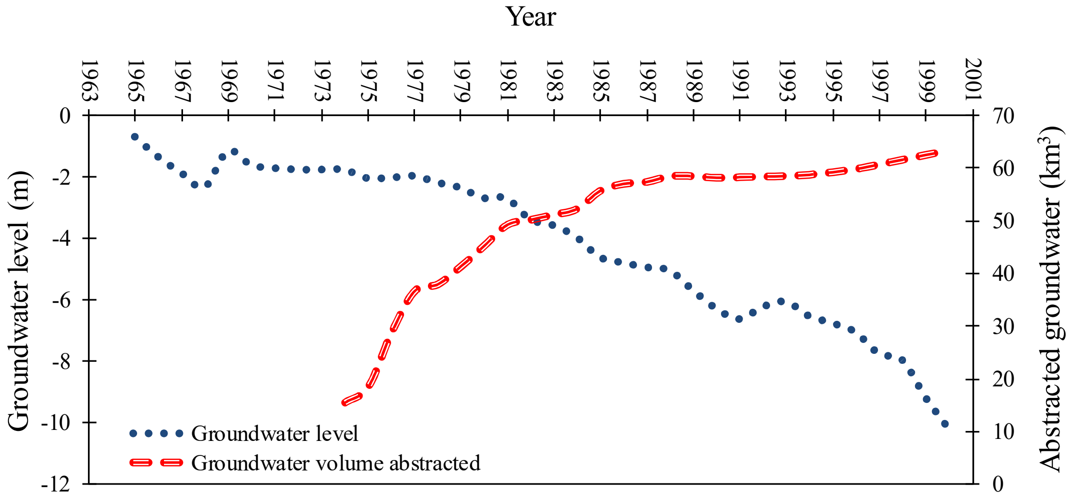

Fig. S1. Changes in average groundwater table (1965-2001) and extracted groundwater volume (1974 to 2001) in Iran.

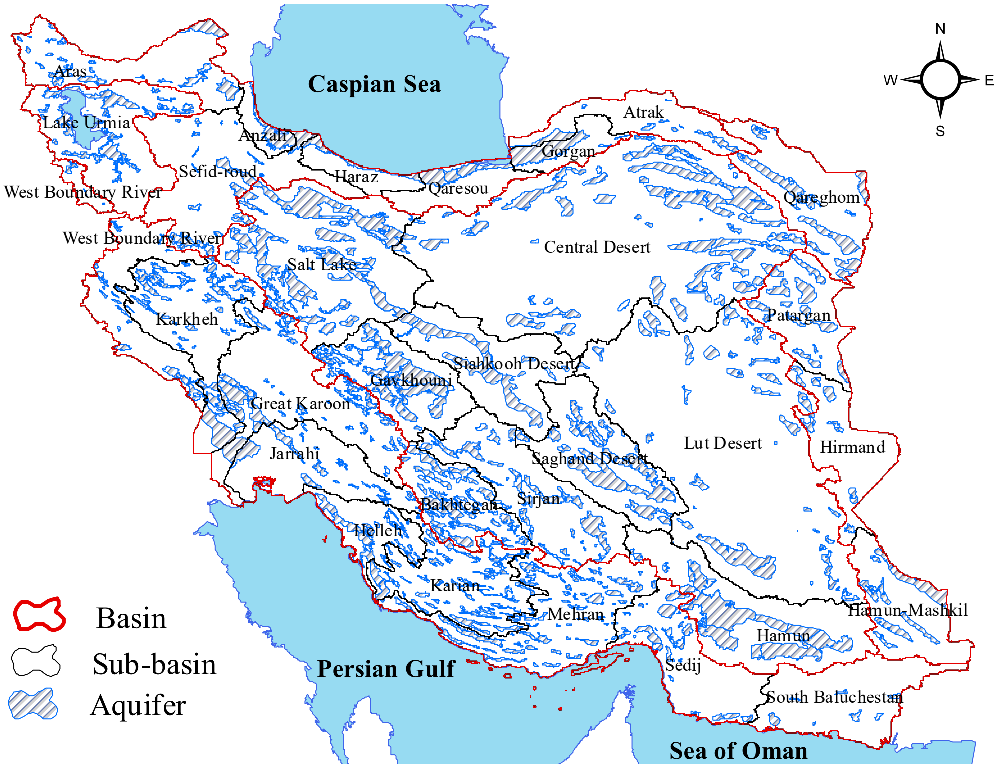

Fig. S2. Major water basins, sub-basins, and aquifers across Iran. Major water basins are: (i) Central Plateau, (ii) Persian Gulf and Sea of Oman, (iii) Caspian Sea, (iv) Eastern Boundary, (v) Lake Urmia, and (vi) Qareghom. Sub-basins are: (i-1) Salt Lake (Daryacheh Namak), (i-2) Gavkhouni (Gavkhuni), (i-3) Bakhtegan, (i-4) Sirjan, (i-5) Hamun, (i-6) Lut Desert, (i-7) Central Desert, (i-8) Siahkooh Deseart, (i-9) Saghand Desert, (ii-1) West Boundary River, (ii-2) Karkheh, (ii-3) Great Karoon (Karoun or Karun), (ii-4) Jarrahi (Jarahi), (ii-5) Helleh, (ii-6) Karian, (ii-7) Mehran, (ii-8) Sedij, (ii-9) South Baluchestan, (iii-1) Aras, (iii-2) Sefid-roud, (iii-3) Haraz, (iii-4) Qaresou, (iii-5) Gorgan, (iii-6) Atrak, (iv-1) Patargan, (iv-2) Hirmand, (iv-3) Hamun-Mashkil, (v-1) Lake Urmia, and (vi-1) Qareghom. Aquifers underly about 260,000 km^2 (i.e., 16%) of Iran's area.

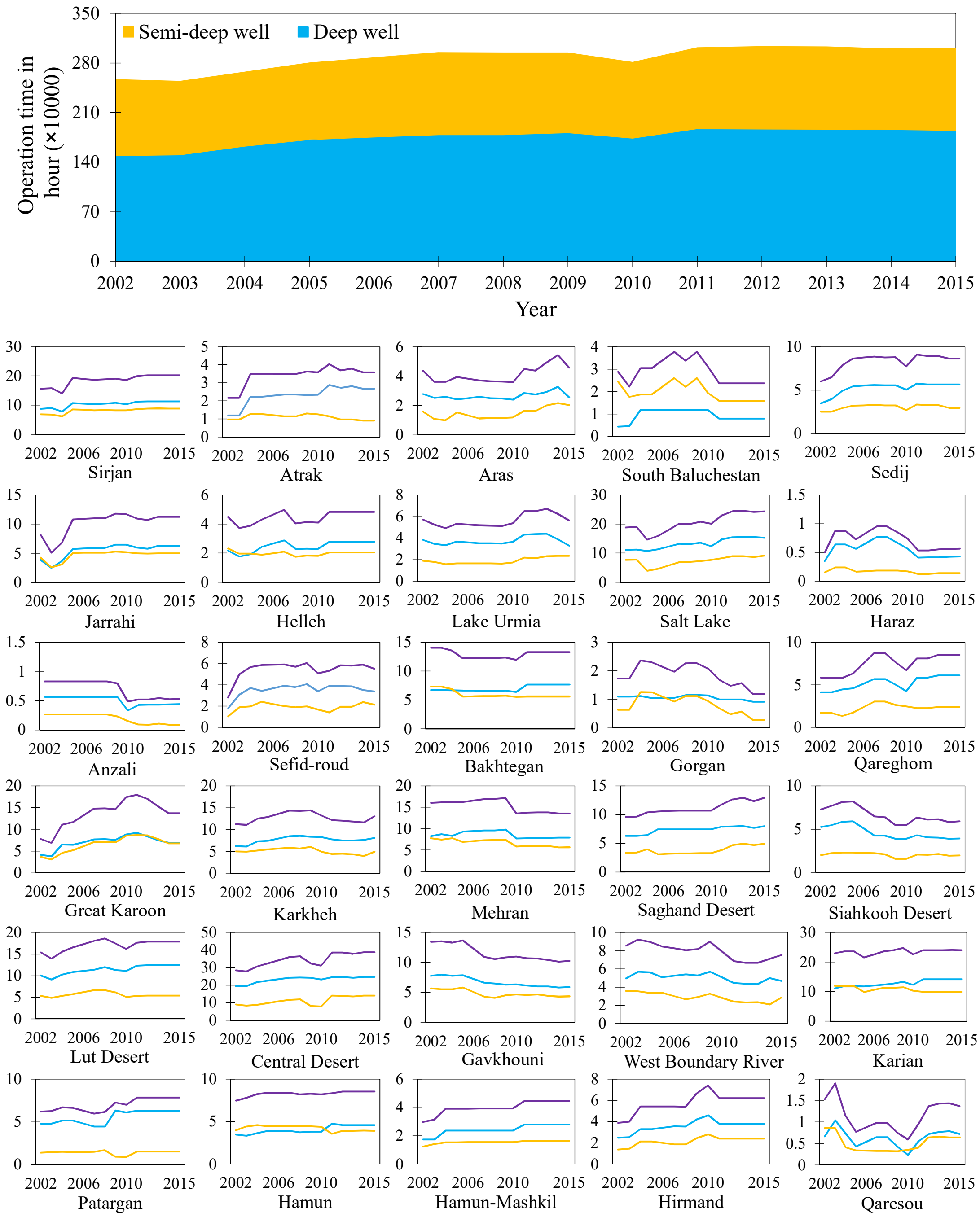

Fig. S3. Operation time (in hour) for semi-deep wells (yellow), deep wells (blue), and all wells (purple) in Iran and in each sub-basin during 2002-2015.

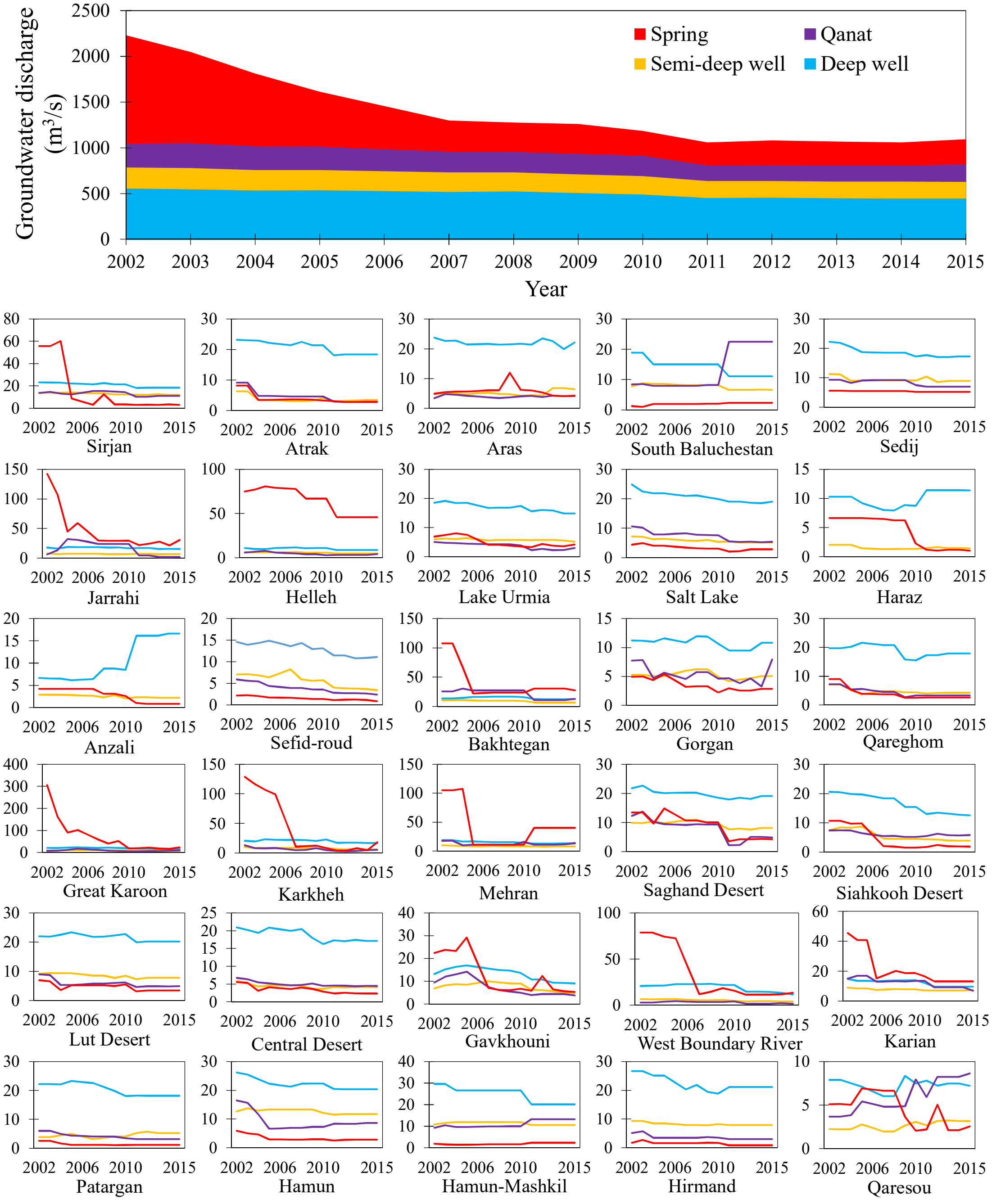

Fig. S4. Groundwater discharge (m^3/s) from deep wells (blue), semi-deep wells (yellow), qanats (purple), and springs (red), in Iran and in each sub-basin during 2002-2015.

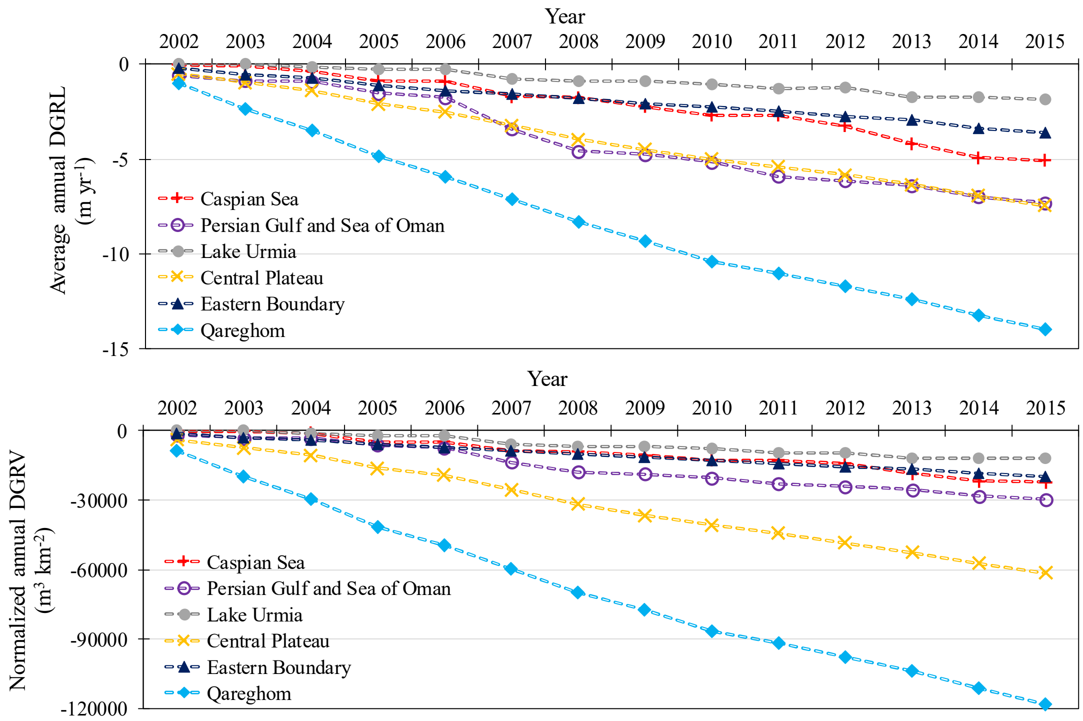

Fig. S5. Cumulative decline in groundwater resource volume (DGRV) and level (DGRL) in six major water basins across Iran from 2002 to 2015.

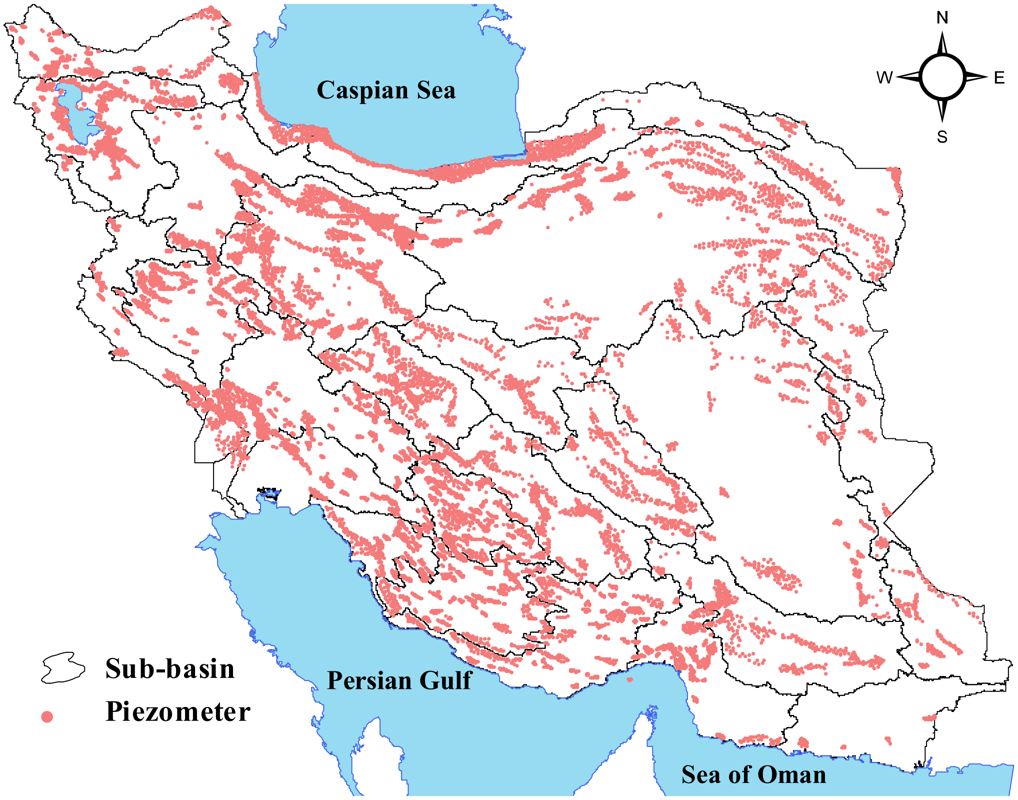

Fig. S6. Spatial distribution of 12,230 piezometers in Iran.

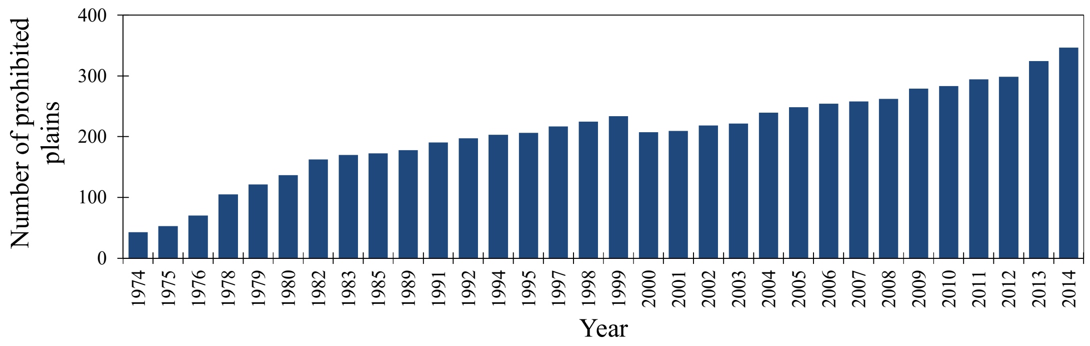

Fig. S7. Increase in the number of "prohibited plains" in Iran (where drilling of new wells is banned except for potable water) due to extensive groundwater table decline during the last five decades.

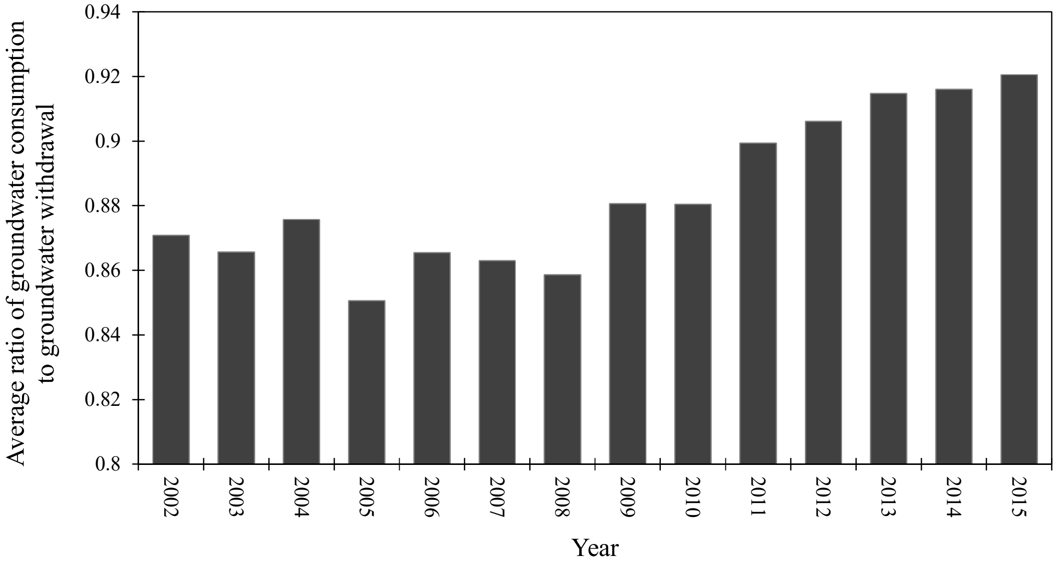

Fig. S8. Average ratio of groundwater consumption to groundwater withdrawal across Iran during 2002–2015.

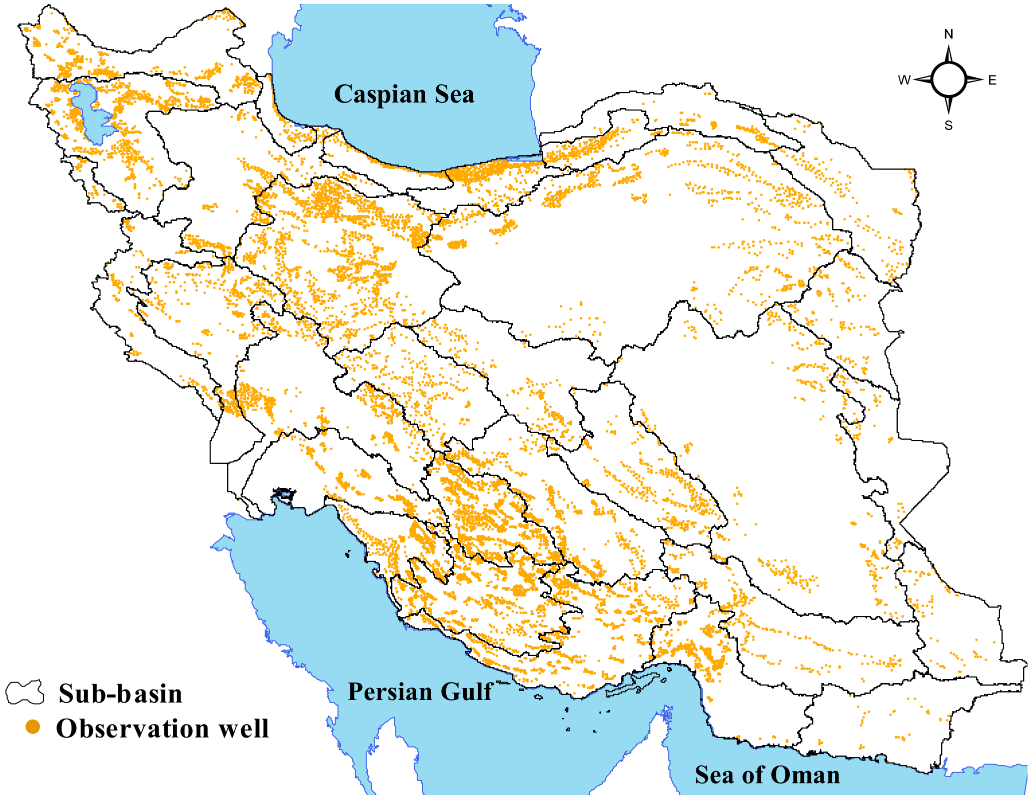

Fig. S9. Spatial distribution of 14,850 observation wells for electrical conductivity (EC) measurement in Iran.

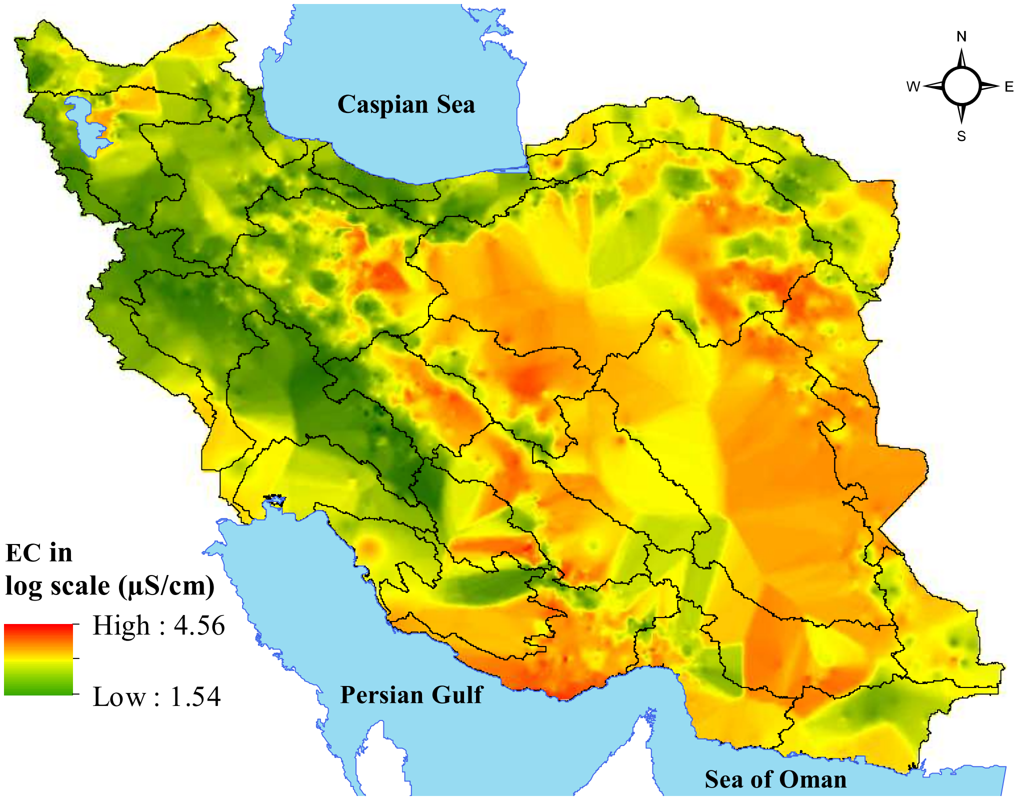

Fig. S10. Spatial distribution of average electrical conductivity (EC) in Iran based on inverse distance weighting method using groundwater EC data from 14,850 observation wells during the study period (2002-2015). The average groundwater EC varied between 34.6 µS/cm and 36,307.8 µS/cm.

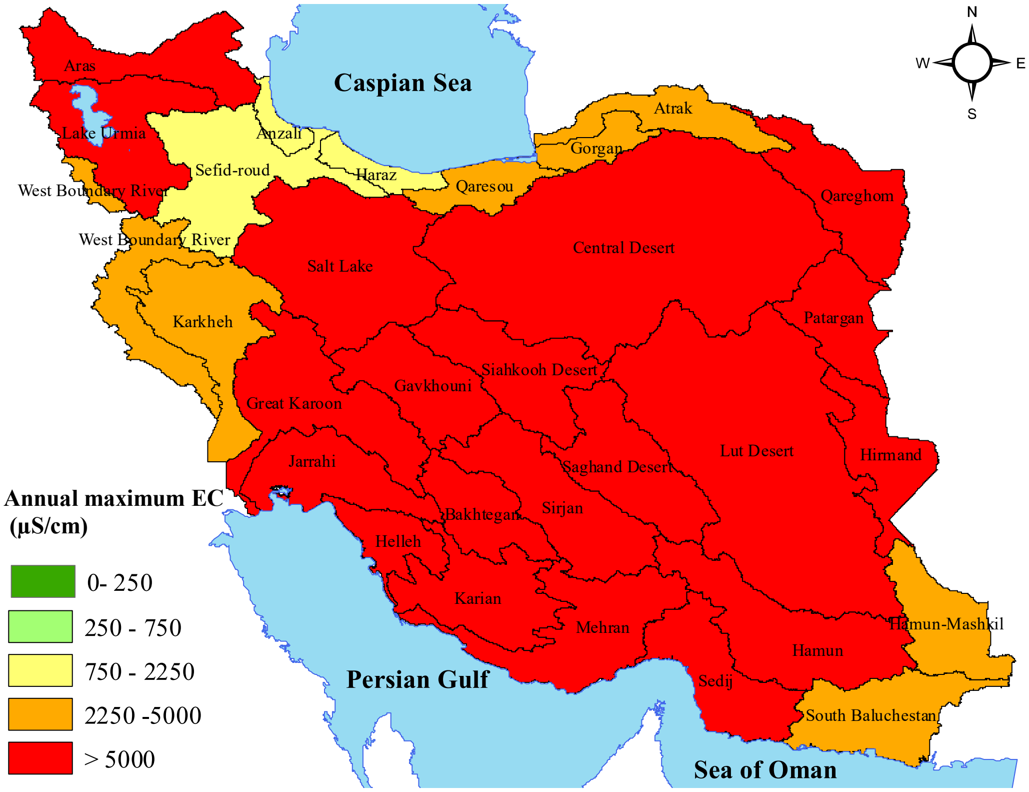

Fig. S11. Spatial distribution of annual maximum electrical conductivity (EC) in sub-basins during 2002–2015. The annual maximum EC in 20 sub-basins exceeded 5000 µS/cm, which is the upper threshold in USSL classification for irrigation of salt-tolerant crops in light soils. The highest EC level was recorded in Mehran and Helleh sub-basins (> 32000 µS/cm) adjacent to the Persian Gulf, indicating likely saltwater intrusion into the coastal aquifers.

Table S1. Annual decline in groundwater resources volume (DGRV) and level (DGRL) in each sub-brain during 2002-2015.

Basin	Sub-basin	Area (km^2)	Annual DGRV (MCM/yr)	Annual DGRL (cm/yr)
Caspian Sea	Aras	39,779	25.45	12.80
	Anzali	7,036	10.06	13.86
	Sefid-roud	59,194	66.33	55.49
	Haraz	10,893	11.01	26.20
	Qaresou	18,775	26.68	24.28
	Gorgan	12,987	118.11	66.64
	Atrak	26,396	21.48	32.23
Persian Gulf and Sea of Oman	West Boundary River	39,298	21.98	31.03
	Karkheh	51,912	136.44	48.63
	Great Karoon	66,676	148.73	73.04
	Jarrahi	40,821	32.64	43.06
	Helleh	21,309	42.15	38.43
	Karian	47,802	141.04	65.19
	Mehran	62,896	234.91	65.44
	Sedij	44,792	135.42	53.64
	South Baluchestan	48,524	1.72	26.07
Lake Urmia	Lake Urmia	51,762	44.77	13.15
Central Plateau	Salt Lake	92,884	1,089.67	96.24
	Gavkhouni	41,552	132.48	32.90
	Bakhtegan	31,452	265.37	82.38
	Sirjan	57,125	319.44	50.92
	Hamun	69,375	184.98	53.23
	Lut Desert	206,354	207.22	33.81
	Central Desert	226,533	902.71	57.45
	Siahkooh Desert	48,599	158.42	23.43
	Saghand Desert	50,737	350.17	64.82
Eastern Boundary	Patargan	33,086	119.85	36.67
	Hirmand	33,590	8.52	9.95
	Hamun-Mashkil	36,508	18.48	30.78
Qareghom	Qareghom	44,296	372.84	99.93
**Iran**		**1,622,943**	**5349.07 MCM**	**49.38 cm/yr (weighted average)**

References

(1) IWRMC (Iran Water Resources Management Company), *Methods and tables for flow measurement*. Iran Ministry of Energy, Iran (1982), (In Persian).

(2) IWRMC (Iran Water Resources Management Company), *A guideline for developing average groundwater hydrograph using the Thiessen Polygon Method*. Iran Ministry of Energy, Report No. 420-60-143, Iran (1990a), (In Persian).

(3) IWRMC (Iran Water Resources Management Company), *A guideline for calculation of monthly and annually groundwater withdrawal from qanats*. Iran Ministry of Energy, Report No. 420-57-135, Iran (1990b), (In Persian).

(4) IWRMC (Iran Water Resources Management Company), *A guideline for determination of location of excavation wells and piezometers in alluvial deposits*. Iran Ministry of Energy, Report No. 420-31-75, Iran (1990c), (In Persian).

(5) IWRMC (Iran Water Resources Management Company), *A guideline for determination of location and maintenance of observation wells*. Iran Ministry of Energy, Report No. 420-30-74, Iran (1990d), (In Persian).

(6) IWRMC (Iran Water Resources Management Company), *Groundwater quality sampling guideline*. Iran Ministry of Energy, Report No. 420-168-527, Iran (1992), (In Persian).

(7) IWRMC (Iran Water Resources Management Company), *A guideline for developing aquifer chemograph using the Thiessen Polygon Method*. Iran Ministry of Energy, Report No. 420-320-1361, Iran (2001), (In Persian).

(8) IWRMC (Iran Water Resources Management Company), *A guideline for selecting groundwater resources (wells, springs, and qanats) and measuring groundwater discharge and withdrawal*. Iran Ministry of Energy, Report No. 420-336-1448, Iran (2002a), (In Persian).

(9) IWRMC (Iran Water Resources Management Company), *A guideline for designing, monitoring, and quality control of groundwater resources*. Iran Ministry of Energy, Report No. 420-325-1424, Iran (2002b), (In Persian).
